# Ablation index-guided high-power ablation for superior vena cava isolation in patients with atrial fibrillation

**DOI:** 10.3389/fcvm.2022.1033297

**Published:** 2022-11-24

**Authors:** Luqian Cui, Shihua Cui, Shujuan Dong, Jingchao Li, Haijia Yu, Huihui Song, Yongmei Han, Yingjie Chu

**Affiliations:** ^1^Department of Cardiology, Henan Provincial People’s Hospital, Zhengzhou, China; ^2^Dalian Medical University, Dalian, China

**Keywords:** ablation index, high-power ablation, superior vena cava isolation, atrial fibrillation, cardiac electrophysiology

## Abstract

**Background:**

The strategy of ablation index (AI)-guided high-power ablation seems to be a novel strategy for performing pulmonary vein isolation (PVI). An AI-guided high-power ablation strategy was used in this study to determine whether superior vena cava isolation (SVCI) after PVI was feasible and safe for patients with AF.

**Methods:**

Data from 53 patients with AF were collected. Mapping and ablation of SVC were performed. The applied power was set at 45 W and the procedure was guided by AI. The SVC was divided into six segments in a cranial view. The RF applications and AI values in different segments were compared and analyzed. Using receiver operating characteristic (ROC) analysis, the diagnostic accuracy of AI value for predicting segment block was evaluated.

**Results:**

Electrical SVCIs were successfully achieved in all patients. SVCI was performed by segment ablation in most cases, with RF applications in different segments. The mean AI value in non-lateral walls was higher than that of the lateral wall (392 ± 28 vs. 371 ± 37, *P* < 0.001). Acutely blocked sites had significantly larger AI values compared with no-blocked sites (390 ± 30 vs. 343 ± 23, *P* < 0.001). The optimal AI cut-off value for non-lateral segments was 379 (sensitivity: 75.9%, specificity: 100%) and for lateral segments was 345 (sensitivity: 82.3%, specificity: 100%).

**Conclusion:**

The AI values were predictive of the acute conduction block of SVCI. With AI values of 345 and 379, respectively, conduction block was achieved in the lateral walls at a lower level than in the non-lateral walls.

## Introduction

In recent years, there has been a growing interest in the high-power ablation strategy (1–6). The ablation index (AI), which weighs contact force (CF), radiofrequency time, and applied power, has been shown to aid in the creation of long-lasting ablation lesions (7–9). There are several studies, which applied AI-guided high-power ablation strategy in atrial fibrillation (AF) ablation and had been validated to improve clinical outcomes after ablation (10–12).

The superior vena cava (SVC) plays an important role in non-pulmonary veins (PVs) foci to trigger AF, and SVC isolation (SVCI) has been demonstrated to reduce AF recurrence in the several reports (13–17). However, because of potential risks, including sinus node injury (SNI), phrenic nerve injury (PNI), and SVC stenosis, there haven’t been optimal ablation strategies for SVCI in previous reports. This study was undertaken to evaluate the AI-guided high-power ablation strategy for SVCI.

## Materials and methods

### Study population

We analyzed a total of 53 objects with paroxysmal AF who received the first ablation operation in our center from September 2020 to August 2021. All objects were sedated using general anesthesia protocol by anesthetists. All patients performed PV isolation (PVI), after which an electrophysiological examination was performed. SVCI was undergone when there are trigger foci Inducing tachycardia or active superior vena cava potentials. No liner ablation was performed except for a related atrial arrhythmia. All patients received cardiac contrast-enhanced Computed tomography or transesophageal echocardiography to exclude left atrial thrombosis. All patients took warfarin or new oral anticoagulants (noacs) for at least 4 weeks and stopped more than five half-lives for all antiarrhythmic medications. The study met the requirements of the Declaration of Helsinki and received approval from the institutional ethics committee. Before any procedures, all patients provided their signed, informed permission.

### Mapping and ablation protocol

After puncturing jugular and femoral veins, under the guidance of X-ray or intracardiac echocardiography, a decapolar catheter was inserted into the coronary sinus, and a single transseptal puncture was made with a Swartz sheath (Abbott, Chicago, USA). After the transseptal puncture, Heparin was given at a dose of 100 U/kg body weight, and it was given again to keep the activated clotting time between 300 and 350 s. Under the direction of an electro-anatomy mapping system (EAM; Biosense Webster, Irvine, USA), mapping and ablation were performed by experienced electrophysiological doctors, using a Pentaray mapping catheter (Biosense Webster, Irvine, USA) and Thermocool Smart touch SF catheter (Biosense Webster, Irvine, USA). Ablation points were marked automatically according to VisiTag (Biosense Webster, Irvine, USA) settings. (The size of the lesion tag was set at 2.5 mm, minimum time 3 s.) Throughout the process, the irrigation flow was 15 ml per minute and the radiofrequency (RF) power was set to 45 W.

### The protocol of superior vena cava isolation

**FIGURE 1:**
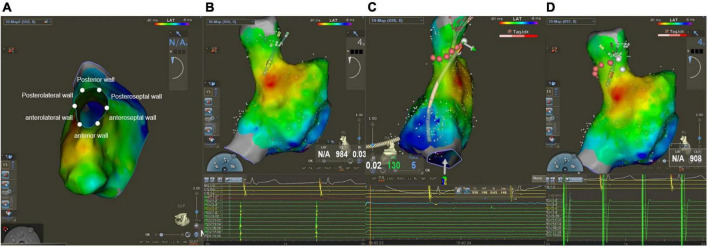
Mapping and ablation of superior vena cava (SVC). **(A)** Anterior, anterolateral, posterolateral, posterior, posteroseptal, and anteroseptal segments in a cranial view. **(B)** Potentials from sinus rhythm in SVC before ablation. **(C)** Absence potential in SVC after ablation. **(D)** Dissociation of SVC potential during pacing.

After finishing PVI, operators used a PentaRay catheter to create Electroanatomical maps of the right atrium (RA) and SVC during sinus rhythm. Besides, an electrophysiological examination was performed at the same time. The SVC was divided into six segments in a cranial view: anterior, anterolateral, posterolateral, posterior, posteroseptal, and anteroseptal portions (Figure 1A). High output pacing (10 mA) was performed at locations on the lateral wall prior to the administration of RF to locate the phrenic nerve and mark its position on the Electroanatomical SVC map. Fluoroscopy showed diaphragmatic movement at the phrenic nerve capture site during the RF delivery. The delivery was immediately halted as soon as PNI was suspected. If the ablation catheter recorded local SVC potentials (Figure 1B), SVCI was performed in the order of the anteroseptal, posteroseptal, posterior, anterior, anterolateral, and then posterolateral segments at the roofline level of the right PVI (generally 10 mm above the RA-SVC junction). Catheter operators used point-by-point ablation and spaced lesions ≤6 mm apart. The AI values were selected at the catheter operator’s discretion based on the achievement of SVCI. RF applications were tagged on the electroanatomic map. A second electrophysiological examination was performed during sinus rhythm after a 20-min waiting period to find conduction gaps or local potentials within segments. Touch-up ablations were carried out in the event that gaps or local potentials were discovered. The endpoint of the SVCI was the bidirectional block and absence or dissociation of potential from SVC until the end of the procedure (Figures 1C,D).

Before, during, and after the ablation procedure, electrocardiogram (ECG) recordings and diaphragmatic movement were monitored. All patients underwent a chest X-ray prior to and the day after the procedure. Phrenic nerve injury was defined as the elevation of the ipsilateral diaphragm under X-ray fluoroscopy and abnormal movement of the diaphragm during inspiratory movement. Sinus node injury was defined as ECG showing an average heart rate of <45 beats/min or sinus arrest >3S. Besides, complications including pericardial effusion, pericardial tamponade, esophagus injury, and cardiovascular ischemic attack had also been recorded.

Patients were scheduled for follow-up at 1, 3, 6, and every 6 months thereafter, or whenever they showed signs of arrhythmia. At each visit, an electrocardiogram (ECG) with 12 leads and a Holter monitoring for 24 h were collected. After a 3-month blanking period, any symptomatic or asymptomatic atrial arrhythmia lasting longer than 30 s was considered a recurrence of AF.

### Statistical analysis

The SPSS 25.0 software from SPSS Inc., was used for all statistical analyses. A Student’s *t*-test was used to compare continuous variables, which are expressed as the mean ± the standard deviation (SD) for normally distributed variables. The Mann–Whitney U test is utilized for group comparisons, and the skew distribution measurement data are expressed in M (Q1, Q3). The Fisher-s exact test or the chi-square test was used to examine categorical variables. A receiver operating characteristic (ROC) curve analysis of AI values in segments with and without block was also carried out. As a measure of diagnostic accuracy, the area under the ROC curve (AUC) was calculated. When a physician determined block and AI values predicted block, this was considered a true positive. Using the Youden Index, the best cut-off values were determined from the ROC analysis. A *p*-value of 0.05 was considered to be statistically significant for each two-tailed test.

## Results

### Patient characteristics

There were 46 patients with paroxysmal AF in whom SVCI, following the PVI, was performed. SVCI wasn’t performed due to the lack of SVC potential in the remaining seven patients. Table 1 displays the characteristics of patients. The average age of the patients was 62.3 ± 11.0 years, and 19 (35.8%) of them were female; the median duration was 3.0 (2.0, 7.0) years; the CHA2DS2-VASc score was 2.0 (1.0, 3.0); the average left atrial diameter (LAD) was 40.0 ± 4.9 mm; the average left ventricular ejection fraction (LVEF) was 63.9 ± 4.7%.

**TABLE 1:** Baseline clinical characteristics (*N* = 53).

Characteristic	Value
Age (y)	62.3 ± 11.0
Sex: female	19 (35.8%)
AF history (y)	3.0 (2.0, 7.0)
CHA2DS2-VASc score	2.0 (1.0, 3.0)
LAD (mm)	40.0 ± 4.9
LVEF (%)	63.9 ± 4.7

AF, atrial fibrillation; LAD, left atrial diameter; LVEF, left ventricular ejection fraction.

### Superior vena cava isolation

The ablation area was divided into six circumferential SVC segments for the regional analysis, as depicted in Figure 1A.

Superior vena cava isolation was performed in 46 of the 53 patients. A total of 45 W power was used exclusively for all ablation lesions. The SVCI’s procedure time was 9.5 ± 4.5 min. With a mean of 7.6 ± 2.9 RF applications, all patients were able to achieve SVCIs. A first pass block was obtained in 251 segments (90.9%). The total number of RF applications until SVC isolation was 353 points.

Table 2 depicts the characteristics of each segment of SVCI lesions. The endpoint of the SVCI could be achieved by segment ablation in most cases. The ablation was performed at the different SVC walls. RF applications were located on the anteroseptal wall in 20 patients (43.5%), posteroseptal wall in 38 patients (82.6%), posterior wall in 40 patients (87.0%), anterior wall in 37 patients (80.4%), anterolateral wall in 27 patients (58.7%), and posterolateral wall in 23 patients (50.0%).

**TABLE 2:** Characteristics of the SVC ablation lesions in different segments.

Segments	Patients (*n* = 46)	RF applications (*n* = 353)	AI values	RF duration (s)	CF (g)	Impedance (Ω)	Voltage (mV)
Anteroseptal	20 (43.5%)	38 (10.8%)	395 ± 25	8 ± 2	11 ± 3	124 ± 8	0.33 ± 0.10
Posteroseptal	38 (82.6%)	74 (21.0%)	392 ± 26	9 ± 2	11 ± 2	126 ± 7	0.33 ± 0.10
Posterior	40 (87.0%)	81 (22.9%)	393 ± 31	10 ± 3	11 ± 3	124 ± 7	0.33 ± 0.09
Anterior	37 (80.4%)	72 (20.4%)	391 ± 28	9 ± 3	12 ± 3	123 ± 7	0.36 ± 0.09
Anterolateral	27 (58.7%)	45 (12.7%)	370 ± 37	7 ± 2	9 ± 2	123 ± 6	0.34 ± 0.08
Posterolateral	23 (50%)	43 (12.2%)	371 ± 38	8 ± 2	9 ± 1	124 ± 6	0.34 ± 0.09

RF, radiofrequency; AI, ablation index; CF, contact force.

**FIGURE 2:**
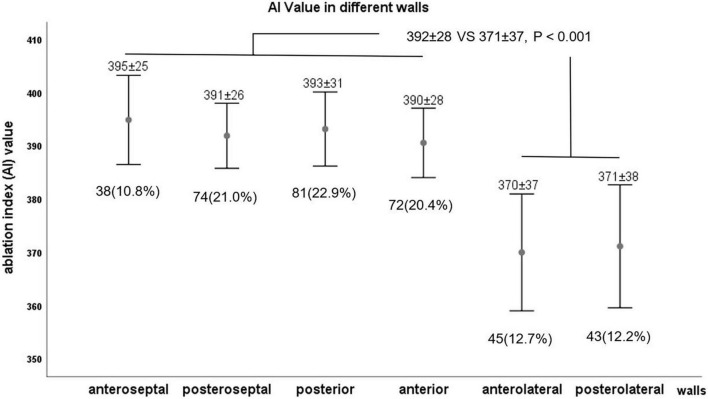
The value of ablation index (AI) in different walls.

The number of RF applications and parallel AI values to eliminate SVC potential was different on different walls, which was shown in Figure 2. RF applications were needed at 38 (10.8%) points (mean AI value of 395 ± 25) in the anteroseptal segment, 74 (21.0%, 391 ± 26) in the posteroseptal segment, 81 (22.9%, 393 ± 31) in the posterior segment, 72 (20.4%, 390 ± 28) in the anterior segment, 45 (12.7%, 370 ± 37) in the anterolateral segment, and 43 (12.2%, 371 ± 38) in the posterolateral segment. It was demonstrated that the mean AI value in septal, posterior, and anterior walls were similar, which was higher than that of the lateral wall (392 ± 28 vs. 371 ± 37, *P* < 0.001).

**FIGURE 3:**
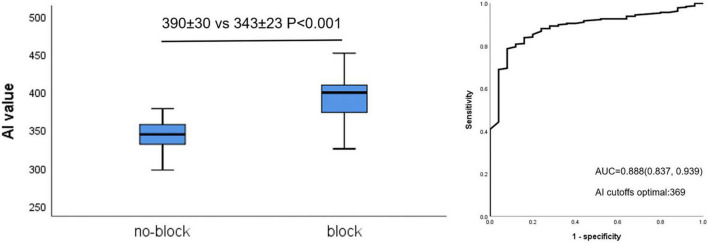
Relationship between AI value and acute block after first-pass encirclement. Mean AI value in acutely blocked and no-blocked sites **(Left)** and corresponding ROC curve analysis **(Right)**.

Figure 3 shows that AI values for acutely blocked sites were significantly larger than for no-blocked sites (390 ± 30 vs. 343 ± 23 *P* < 0.001, left). The optimal AI cut-off value (Youden Index) was determined by ROC analysis at 369 (sensitivity: 78.7%, specificity: 92.0% Figure 3, right). The AUC was 0.888.

**FIGURE 4:**
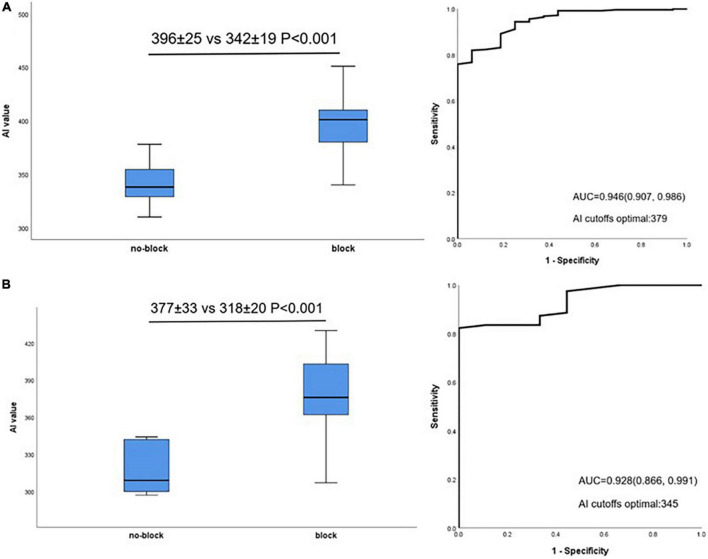
Relationship between AI value and acute block after first-pass encirclement. **(A)** Mean AI value in non-lateral segments acutely blocked and no-blocked sites (Left) and corresponding ROC curve analysis (Right). **(B)** Mean AI value in lateral segments acutely blocked and no-blocked sites (Left) and corresponding ROC curve analysis (Right).

The relationship between mean AI value and acutely block was further evaluated in lateral and non-lateral segments. Acutely blocked sites had significantly larger mean AI compared with no-blocked sites in non-lateral segments (396 ± 25 vs. 342 ± 19 *P* < 0.001, Figure 4A, left). Similar to this, in lateral segments, acutely blocked sites exhibited much higher mean AI than no-blocked sites (377 ± 33 vs. 318 ± 20 *P* < 0.001, Figure 4B, left). Non-lateral segments had an optimal AI cut-off value of 379 (sensitivity: 75.9%, specificity: 100%, Figure 4A, right), while lateral segments had an optimal cut-off value of 345 (sensitivity: 82.3%, specificity: 100%, Figure 4B, right).

There were no complications in any patients, including SNI and PNI, which were related to the procedure of SVCI. Every patient was followed up for at least 6 months, There were three patients with AF recurrence documented by ECG or holter, one of whom had atrial tachycardia and received the second ablation. It is the reconnection of the right PV that contributed to the occurrence of arrhythmia. The AF-free survival rate at 6 months was 94.3%.

## Discussion

To the best of our knowledge, the AI-guided high-power ablation technique for SVCI is being introduced for the first time in our study. In all patients, an electrical SVCI was achieved without compromising the risk of complications. The majority of cases involved segment ablation for SVCI. The acute block of SVCI could be predicted by the AI values applied during ablation. In comparison to the non-lateral segments, the conduction block could be achieved with a significantly lower AI value in the lateral segments. Optimal AI values were 379 for the non-lateral segments and 345 for the lateral walls.

### Superior vena cava isolation

In the past two decades, catheter ablation has been well-developed and is the mainstream method for the treatment of AF. PVI has become the foundation of AF ablation because ectopic beats in the PVs are the most common cause of AF (18). However, the outcome of a PVI alone is unsatisfactory. Therefore, In order to improve the outcome of AF ablation, it is necessary to look for new ablation techniques in addition to PVI. It was reported that AF was also triggered and maintained by non-PV ectopic beats, which could come from the crista terminalis, coronary sinus ostium, or SVC, among other places (13–15). After the first ablation, mapping and ablating non-PV AF triggers were also essential for AF freedom.

Superior vena cava was reported to be the most common non-PVs foci to trigger AF, the incidence of which was 5.3–12.0% (13–17). Therefore, in addition to the PVI, it was anticipated that the SVCI would be helpful in enhancing the clinical outcomes of AF ablation. However, there weren’t all studies in support of this (19). We consider that performing SVCI has limited effect and is controversial for the reason that due to the close proximity of the sinus node and phrenic nerve, it is challenging to create a lasting lesion around the SVC (20, 21).

### Ablation index-guided high-power ablation

The quality of RF lesions was recognized as a major determinant of arrhythmias recurrence. The objective of ablation is to produce transmural lesions while minimizing collateral damage to important structures. A high-power ablation strategy for PVI has been demonstrated to create contiguous and durable RF lesions without compromise on complications in several studies (1–6). The delivered energy causes lesions through a combination of resistive and conductive heating phases. While the latter conducts heating to deeper tissue, the former causes local, immediate tissue injury (22). A high-power ablation strategy makes it possible to deliver the same amount of energy in a shorter ablation time, which reduces conductive heating to limit collateral damages and increases local resistive heating to create transmural lesions (23).

However, the atrial myocardium extending into the SVC is thinner than that of PVs (24). Besides, the right phrenic nerve runs close to the lateral wall of the SVC and the ablation sites are near the sinus node (20, 21). Considering the above reasons, an optimal setting of RF energy delivered was needed to ensure the efficacy and safety of SVCI. AI as an ideal parameter for PVI showed a high single procedure success in several clinical studies (7–9). In a recent paper, Kawano et al. examined the SVCI’s target AI value for the first time and revealed that 350 may be the SVCI’s target AI value, and this information could be helpful to the safety and efficacy of SVCI (25). In our study, the AI value is bigger and the reasons may be the followings. Firstly, the applied power was higher in our study. Secondly, the level of the ablation line and endpoint of each lesion were different.

Ablation index-guided high-power ablation strategy is a combined ablation technique, which incorporates ideal ablation parameters into high-power ablation and makes the ablation procedure safer and more effective (10–12). On one hand, it uses high-power to achieve transmural lesions; on the other hand, limits the collateral damages. Moreover, the strategy may be an optimal ablation technique for SVCI.

In all patients, an electrical SVCI was achieved in our studies. What’s more, the AI-guided high-power ablation for SVCI seemed not to be associated with additionally increased complications, including SNI and phrenic nerve injury. The SVCI time was 9.5 ± 4.5 min. During the waiting period for PV reconnections following the CPVI, which has been reported to be very important for achieving a durable PVI, we carried out an empiric SVCI (26). As a result, the SVCI’s procedural time was not wasteful or time-consuming.

It was reported that the myocardial sleeves extending into the SVC were discontinuous in most cases. Besides, the RA-SVC myocardial connection varied in thickness and length (24). In our study, more RF applications were located on septal, posterior, and anterior walls, and in which higher AI value was needed to achieve the electrical SVCI. The following reasons may account for this fact. Firstly, the myocardial sleeves in the septal, posterior, and anterior walls were thicker. Secondly, the lateral wall had a potential risk of SNI or PNI, therefore, the performer used a lower AI value in this area.

Ablation index-guided high-power ablation with different target AI values for lateral and non-lateral regions may increase the likelihood of successful acute SVCI, given that AI value has been shown in this study to be predictive of the acute block. The non-lateral segments’ optimal values were 379, while the lateral walls’ optimal values were 345, providing the best balance between safety and efficacy. However, concerns regarding efficacy and safety differ between RA regions, with the non-lateral wall having a lower risk of extra-cardiac injury. In order to evaluate this strategy’s safety and efficacy, a future prospective study with large sample size is required to validate these suggested target values.

### Study limitations

This was a small study with a single center and a small sample. To determine whether AI-guided high-power ablation for SVCI is safe and effective, large-scale research is required. The optimal AI values that this study found pertain to acute conduction block and may not result in the formation of lasting lesions. Due to the brief follow-up period, neither the SVC stenosis nor the long-term efficacy was thoroughly evaluated.

## Conclusion

In patients with paroxysmal AF, The AI values during ablation were predictive of the acute block of SVCI. The lateral walls required smaller AI values for conduction block compared to the non-lateral walls, with AI values of 345 and 379, respectively.

## Data availability statement

The original contributions presented in this study are included in the article/supplementary material, further inquiries can be directed to the corresponding authors.

## Ethics statement

The studies involving human participants were reviewed and approved by Ethics Committee of Henan Provincial People’s Hospital. The patients/participants provided their written informed consent to participate in this study.

## Author contributions

LC, SD, and YC designed the study. LC and SC collected and analyzed the data statistically. All authors contributed to the writing of this manuscript.

## References

[1] Vassallo F Cunha C Serpa E Meigre LL Carloni H Simoes A Jr Comparison of high-power short-duration (HPSD) ablation of atrial fibrillation using a contact force-sensing catheter and conventional technique: initial results. *J Cardiovasc Electrophysiol.* (2019) 30:1877–83. 10.1111/jce.14110 31397522

[2] Okamatsu H Koyama J Sakai Y Negishi K Hayashi K Tsurugi T High-power application is associated with shorter procedure time and higher rate of first-pass pulmonary vein isolation in ablation index-guided atrial fibrillation ablation. *J Cardiovasc Electrophysiol.* (2019) 30:2751–8. 31600006 10.1111/jce.14223

[3] Ejima K Higuchi S Yazaki K Kataoka S Yagishita D Kanai M Comparison of high-power and conventional-power radiofrequency energy deliveries in pulmonary vein isolation using unipolar signal modification as a local endpoint. *J Cardiovasc Electrophysiol.* (2020) 31:1702–8. 10.1111/jce.14532 32378266 PMC7383605

[4] Cui L Chu Y Han Y Dong S. Comparison of higher-power and conventional power ablation of atrial fibrillation using contact force-sensing catheters: a systematic review and meta-analysis. *J Interv Card Electrophysiol.* (2021) 62:1–7. 10.1007/s10840-021-00975-3 33730302

[5] Vassallo F Meigre LL Serpa E Cunha C Simoes A Jr Carloni H Changes and impacts in early recurrences after atrial fibrillation ablation in contact force era: comparison of high-power short-duration with conventional technique-FIRST experience data. *J Interv Card Electrophysiol.* (2021) 62:363–71. 10.1007/s10840-020-00911-x 33151444

[6] Hijioka N Kaneshiro T Nehashi T Amami K Nodera M Yamada S Procedural characteristics of pulmonary vein isolation with high-power short-duration setting compared to conventional setting. *BMC Cardiovasc Disord.* (2022) 22:14. 10.1186/s12872-022-02459-2 35067224 PMC8785467

[7] Solimene F Schillaci V Shopova G Urraro F Arestia A Iuliano A Safety and efficacy of atrial fibrillation ablation guided by ablation index module. *J Interv Card Electrophysiol.* (2019) 54:9–15. 10.1007/s10840-018-0420-5 30058055

[8] Ioannou A Papageorgiou N Lim WY Wongwarawipat T Hunter RJ Dhillon G Efficacy and safety of ablation index-guided catheter ablation for atrial fibrillation: an updated meta-analysis. *Europace.* (2020) 22:1659–71. 10.1093/europace/euaa224 32862230

[9] Lepillier A Strisciuglio T De Ruvo E Scaglione M Anselmino M Sebag FA Impact of ablation index settings on pulmonary vein reconnection. *J Interv Card Electrophysiol.* (2022) 63:133–42. 10.1007/s10840-021-00944-w 33570717

[10] Otsuka N Okumura Y Kuorkawa S Nagashima K Wakamatsu Y Hayashida S Actual tissue temperature during ablation index-guided high-power short-duration ablation versus standard ablation: implications in terms of the efficacy and safety of atrial fibrillation ablation. *J Cardiovasc Electrophysiol.* (2022) 33:55–63. 10.1111/jce.15282 34713525

[11] Lee SR Park HS Choi EK Lee E Oh S. Acute and long-term efficacy of ablation index-guided higher power shorter duration ablation in patients with atrial fibrillation: a prospective registry. *J Arrhythm.* (2021) 37:1250–9. 10.1002/joa3.12605 34621423 PMC8485805

[12] Zanchi S Chen S Bordignon S Bianchini L Tohoku S Bologna F Ablation Index-guided high-power (50 W) short-duration for left atrial anterior and roofline ablation: feasibility, procedural data, and lesion analysis (AI high-power linear ablation). *J Cardiovasc Electrophysiol.* (2021) 32:984–93. 10.1111/jce.14973 33634549

[13] Lin WS Tai CT Hsieh MH Tsai CF Lin YK Tsao HM Catheter ablation of paroxysmal atrial fibrillation initiated by non-pulmonary vein ectopy. *Circulation.* (2003) 107:3176–83. 12821558 10.1161/01.CIR.0000074206.52056.2D

[14] Takigawa M Takahashi A Kuwahara T Okubo K Takahashi Y Nakashima E Impact of non-pulmonary vein foci on the outcome of the second session of catheter ablation for paroxysmal atrial fibrillation. *J Cardiovasc Electrophysiol.* (2015) 26:739–46. 10.1111/jce.12681 25845757

[15] Tsai CF Tai CT Hsieh MH Lin WS Yu WC Ueng KC Initiation of atrial fibrillation by ectopic beats originating from the superior vena cava: electrophysiological characteristics and results of radiofrequency ablation. *Circulation.* (2000) 102:67–74. 10880417 10.1161/01.cir.102.1.67

[16] Corrado A Bonso A Madalosso M Rossillo A Themistoclakis S Di Biase L Impact of systematic isolation of superior vena cava in addition to pulmonary vein antrum isolation on the outcome of paroxysmal, persistent, and permanent atrial fibrillation ablation: results from a randomized study. *J Cardiovasc Electrophysiol.* (2010) 21:1–5. 10.1111/j.1540-8167.2009.01577.x 19732237

[17] Ejima K Kato K Iwanami Y Henmi R Yagishita D Manaka T Impact of an empiric isolation of the superior vena cava in addition to circumferential pulmonary vein isolation on the outcome of paroxysmal atrial fibrillation ablation. *Am J Cardiol.* (2015) 116:1711–6. 10.1016/j.amjcard.2015.09.005 26434513

[18] Haïssaguerre M Jaïs P Shah DC Takahashi A Hocini M Quiniou G Spontaneous initiation of atrial fibrillation by ectopic beats originating in the pulmonary veins. *N Engl J Med.* (1998) 339:659–66. 9725923 10.1056/NEJM199809033391003

[19] Wang XH Liu X Sun YM Shi HF Zhou L Gu JN. Pulmonary vein isolation combined with superior vena cava isolation for atrial fibrillation ablation: a prospective randomized study. *Europace.* (2008) 10:600–5. 18442966 10.1093/europace/eun077

[20] Chen G Dong JZ Liu XP Zhang XY Long DY Sang CH Sinus node injury as a result of superior vena cava isolation during catheter ablation for atrial fibrillation and atrial flutter. *Pacing Clin Electrophysiol.* (2011) 34:163–70. 10.1111/j.1540-8159.2010.02903.x 20883509

[21] Bai R Patel D Di Biase L Fahmy TS Kozeluhova M Prasad S Phrenic nerve injury after catheter ablation: should we worry about this complication? *J Cardiovasc Electrophysiol.* (2006) 17:944–8. 10.1111/j.1540-8167.2006.00536.x 16800858

[22] Haines DE. The biophysics of radiofrequency catheter ablation in the heart: the importance of temperature monitoring. *Pacing Clin Electrophysiol.* (1993) 16:586–91. 7681962 10.1111/j.1540-8159.1993.tb01630.x

[23] Bhaskaran A Chik W Pouliopoulos J Nalliah C Qian P Barry T Five seconds of 50-60 W radio frequency atrial ablations were transmural and safe: an *in vitro* mechanistic assessment and force-controlled *in vivo* validation. *Europace.* (2017) 19:874–80. 10.1093/europace/euw077 27207815

[24] Kholova I Kautzner J. Morphology of atrial myocardial extensions into human caval veins: a postmortem study in patients with and without atrial fibrillation. *Circulation.* (2004) 110:483–8. 10.1161/01.CIR.0000137117.87589.88 15277325

[25] Kawano D Mori H Tsutsui K Ikeda Y Yamaga M Kawai A The target ablation index values for electrical isolation of the superior vena cava. *J Interv Card Electrophysiol.* (2022) 64:687–94. 10.1007/s10840-021-01112-w 35112239

[26] Wang XH Liu X Sun YM Gu JN Shi HF Zhou L Early identification and treatment of PV re-connections: role of observation time and impact on clinical results of atrial fibrillation ablation. *Europace.* (2007) 9:481–6. 10.1093/europace/eum101 17522081

